# Supplementary material for: Quality of care and post-discharge morbidity among children diagnosed with severe malaria in rural Uganda: A prospective cohort study
Source: PLOS Glob Public Health. 2024 Oct 7;4(10):e0003794. doi: 10.1371/journal.pgph.0003794 (PMC11458001; doi:10.1371/journal.pgph.0003794)
Supplement: S1 Appendix — (PDF) [file pgph.0003794.s002.pdf]

Eligibility & Enrollment Form

REDCap #	______________________ (Automatic Pull - RedCap #)
Date of Screening	______________________
Did child have positive malaria test (RDT or Microscopy)	◯ Yes<br>◯ No<br>◯ No, but the child tested positive at another health center<br>◯ No, due to malaria test stockout, but the clinician suspects malaria
What is the age of the child in years? (Put 0 for children under 1 year old)	______________________
Clinically Eligible (RMSF)	
Not Clinically Eligible - Stop Here (RMSF)	
ELIGIBLE - child is under 18 years, positive malaria test, and consenting parent or guardian	◯ Yes<br>◯ No
Date of Consent of Parent or Guardian	______________________ (Date of consent of parent or guardian)
RA Documenting Consent	◯ Kebaba Georget<br>◯ Kule Herbert<br>◯ Mumbere Samuel<br>◯ Jennifer Kniss<br>◯ Other
Enter name of RA documenting consent	______________________
Time of enrollment	______________________
Preferred contact method	☐ Phone<br>☐ Email<br>☐ Other
If other, please describe:	______________________
Phone number	______________________ (99 if unavailable)
Email	______________________

Sub-county of residence	◯ Bugoye<br>◯ Bwera<br>◯ Ihandiro<br>◯ Karambi<br>◯ Karusandara<br>◯ Kasese Tc<br>◯ Katwe<br>◯ Kabatoro Tc<br>◯ Kilembe<br>◯ Kisinga<br>◯ Kitholhu<br>◯ Kitswamba<br>◯ Kyabarungira<br>◯ Kyarumba<br>◯ Kyondo L.<br>◯ Katwe<br>◯ Mahango<br>◯ Maliba<br>◯ Muhokya<br>◯ Munkunyu<br>◯ Nyakiyumbu<br>◯ Rukoki<br>◯ Other
If other, please state the subcounty you live in.	__________________________________
Study Number<br><br>First Two Letters of Subcounty - Last 2 Digits of Year of Birth - Day of the Month of Enrollment - RedCap Number from above<br><br>Example: Child from Maliba born in 2015, enrolled on the 23rd day of the month, with a RedCap number of 1= MA15231<br><br>*If day of enrollment is less than 10th of the month, put a 0 in front of the number. Example: enter 06 for 6th day of month of enrollment.	__________________________________
Comments	____
Patient is eligible and enrolled	

Demographics Household And Malaria

Research Assistant entering data	◯ Kule Herbert<br>◯ Mumbere Samuel<br>◯ Kebaba Georget<br>◯ Jennifer Kniss<br>◯ Other
What was the name of the research assistant collecting the demographic data?	__________________________
What is the full name of the child?	__________________________
What is the child's age (years)?<br><br>IF CHILD IS LESS THAN 1 YEAR, ENTER 0 WHICH WILL OPEN NEW QUESTION ABOUT MONTHS	__________________________
How many months old is the child?	__________________________<br>(Age in months, child under 1 year old)
What is the child's sex?	◯ Female<br>◯ Male
Is the child enrolled in school?	◯ Yes<br>◯ No, the child is too young<br>◯ No
At what level of school is the child currently enrolled?	◯ Primary school<br>◯ Secondary school<br>◯ Other
What is the ethnic group of the child?	◯ Mukonjo<br>◯ Baganda<br>◯ Banyankore<br>◯ Iteso<br>◯ Lugbara/Madi<br>◯ Basoga<br>◯ Langi<br>◯ Bakiga<br>◯ Karimojong<br>◯ Acholi<br>◯ Bagisu/Sabiny<br>◯ Alur/Jopadhola<br>◯ Banyoro<br>◯ Batoro<br>◯ Other
If other ethnic group is selected, please specify:	________________________________

What is your preferred religion?	◯ Catholic<br>◯ Anglican/Protestant<br>◯ Seventh Day Adventist<br>◯ Pentecostal<br>◯ Other Christian<br>◯ Moslem<br>◯ Bahai<br>◯ Traditional<br>◯ Hindu<br>◯ None<br>◯ Other<br>(Religion of caregiver)
If other religion, please specify	__________________________________

Malaria Prevention and Care Seeking

Insectide Treated Nets

Does your household have any mosquito nets?	◯ Yes<br>◯ No
How many mosquito nets does your household have?	____________________________
How many mosquito nets were used last night?	____________________________
How many household members slept under a mosquito net last night?	____________________________
Did any household members NOT sleep under a mosquito net last night?	◯ Yes<br>◯ No<br>(Members who did not sleep under itn)
Please list the age and sex of any household members who did NOT sleep under a mosquito net last night	__________________________________<br>(Age and sex of members who did not sleep under itn)
Why did these members not sleep under a net?	☐ Not enough nets available<br>☐ Net has too many holes or is damaged<br>☐ Too hot, uncomfortable, or don't like smell<br>☐ No place to hang or place it<br>☐ Not many mosquitoes / Low risk for malaria<br>☐ Other<br>(Select all that apply)

If other reasons stated for not using a mosquito net, please list here:	__________________________________

Indoor Residual Spraying (IRS)

At any time in the past 6 months, has anyone come into your dwelling to spray the interior walls against mosquitoes?	◯ Yes<br>◯ No
Who sprayed the dwelling?<br><br>PROBE FOR ANY OTHERS. RECORD ALL MENTIONED.	◯ Government worker/program<br>◯ Private company<br>◯ Nongovernmental Organization (NGO)<br>◯ Other<br>◯ Don't know
If other sprayer source mentioned, specify here.	__________________________________
Did you pay for your dwelling to be sprayed?	◯ Yes<br>◯ No
If you paid to have you dwelling sprayed, how much did you pay?	____________________________<br>(Shillings)

Malaria Care Seeking

If a child in the household had a fever, where would the caregiver normally take the child for care?	◯ Government health center<br>◯ Private health center<br>◯ Drug shop<br>◯ VHT<br>◯ Traditional healer (i.e. herbs)<br>◯ Clinic<br>◯ Other
If the participant would seek care elsewhere, please specify:	__________________________________
In the last three months, have you taken your child to any of the following for fever?	☐ Government health center<br>☐ Private health center<br>☐ Drug shop<br>☐ VHT<br>☐ Traditional healer (i.e. herbs)<br>☐ Clinic<br>☐ Other<br>(Check all that apply)
If care was pursued elsewhere, please specify:	__________________________________
In the last year has any child in this household been admitted (stayed overnight) to a health clinic or hospital for malaria other than the current stay?	◯ Yes<br>◯ No<br>(Any child admitted in last year?)

In the last year, has any child in this household received intravenous medication (i.e. given through a vein) for malaria	◯ Yes<br>◯ No
If the child has received IV medication for malaria, where did they receive the medicine?	◯ Hospital<br>◯ Clinic<br>◯ Drug Shop<br>◯ Other
If the child received IV medication elsewhere, please specify:	__________________________________
Has any child from this household ever died from malaria?	◯ Yes<br>◯ No
If a child has died from malaria, how old was the child at the time of death? (years)<br><br>If the child is under 1 year old, you place a 0 and a question will ask how many months old the child was.	____________________________
If the child was under 1 year old at the time of death, how many months old was the child?	____________________________

Household Members

Include any individuals who spend most nights of the week in the house.

How many individuals live in this household?	____________________________
Of those individuals, how many adults (age ≥ 18 years) live in this household?	____________________________
How many children (under 18 years old) live in this household?	____________________________
Of those children, how many (age less than 5 years) live in this household?	____________________________

Adult Household Members

Ask information about adult members of the household. The male and female head-of-household (i.e. husband/wife or father/mother) should be Adult #1 and Adult #2. Adult children (age ≥18 years) of the head-of-household should also be included here if they still live in the house.

Questions are available for up to six adult household members. If more than six adults live in the household, then record information about six, then document additional information in Comments section at bottom of form.

Sex of Adult #1	◯ Female<br>◯ Male
Age of Adult #1	______________________<br>(Years)
What category best describes Adult #1	◯ Mother<br>◯ Father<br>◯ Adult child<br>◯ Grandmother<br>◯ Grandfather<br>◯ Other
If other, please specify:	______________________
What is the highest level of school Adult #1 completed?	◯ No school<br>◯ Primary School<br>◯ Secondary School<br>◯ University
What is the marital status of Adult #1?	◯ Unmarried<br>◯ Married<br>◯ Divorced<br>◯ Widowed
Sex of Adult #2	◯ Female<br>◯ Male
Age of Adult #2	______________________<br>(Years)
What category best describes Adult #2	◯ Mother<br>◯ Father<br>◯ Adult child<br>◯ Grandmother<br>◯ Grandfather<br>◯ Other
If other, please specify:	______________________
What is the highest level of school Adult #2 completed?	◯ No school<br>◯ Primary School<br>◯ Secondary School<br>◯ University
What is the marital status of Adult #2?	◯ Unmarried<br>◯ Married<br>◯ Divorced<br>◯ Widowed
Sex of Adult #3	◯ Female<br>◯ Male

Age of Adult #3	______________________ (Years)
What category best describes Adult #3	◯ Mother<br>◯ Father<br>◯ Adult child<br>◯ Grandmother<br>◯ Grandfather<br>◯ Other
If other, please specify:	______________________
Sex of Adult #4	◯ Female<br>◯ Male
Age of Adult #4	______________________ (Years)
What category best describes Adult #4	◯ Mother<br>◯ Father<br>◯ Adult child<br>◯ Grandmother<br>◯ Grandfather<br>◯ Other
If other, please specify:	______________________
Sex of Adult #5	◯ Female<br>◯ Male
Age of Adult #5	______________________ (Years)
What category best describes Adult #5	◯ Mother<br>◯ Father<br>◯ Adult child<br>◯ Grandmother<br>◯ Grandfather<br>◯ Other
If other, please specify:	______________________
Sex of Adult #6	◯ Female<br>◯ Male
Age of Adult #6	______________________ (Years)

What category best describes Adult #6	◯ Mother<br>◯ Father<br>◯ Adult child<br>◯ Grandmother<br>◯ Grandfather<br>◯ Other
If other, please specify:	______________________________
Additional Comments	______________________________

Child Information

Include information on all children under 18 years of age who live in the household. Enter data from oldest child to youngest child. For example, Child #1 should be the oldest child that is under 18 years old.

If more than 6 children live in the household, document additional information in the Comments section below.

Sex of Child #1 (Oldest)	◯ Female<br>◯ Male
Age of Child #1<br><br>IF CHILD IS LESS THAN 1 YEAR, ENTER 0 WHICH WILL OPEN NEW QUESTION ABOUT MONTHS	______________________________<br>(Years)
If Child #1 is less than 1 year of age, how many months old is the child	______________________________<br>(Months)
What category best describes Child #1	◯ Sibling<br>◯ Patient<br>◯ Other
Where was Child #1 born?	◯ Home<br>◯ Government Health Facility<br>◯ Government Hospital<br>◯ Private Clinic or Hospital<br>◯ Other
If other, please list here:	______________________________
Did Child #1 sleep under a bed net last night?	◯ Yes<br>◯ No
Has Child #1 stayed overnight in a hospital or clinic for malaria in the past year?	◯ Yes<br>◯ No

Sex of Child #2	◯ Female<br>◯ Male
Age of Child #2<br><br>IF CHILD IS LESS THAN 1 YEAR, ENTER 0 WHICH WILL OPEN NEW QUESTION ABOUT MONTHS	______________________<br>(Years)
If Child #2 is less than 1 year of age, how many months old is the child	______________________<br>(Months)
What category best describes Child #2	◯ Sibling<br>◯ Patient<br>◯ Other
Where was Child #2 born?	◯ Home<br>◯ Government Health Facility<br>◯ Government Hospital<br>◯ Private Clinic or Hospital<br>◯ Other
If other, please list here:	____________________________
Did Child #2 sleep under a bed net last night?	◯ Yes<br>◯ No
Has Child #2 stayed overnight in a hospital or clinic for malaria in the past year?	◯ Yes<br>◯ No
Sex of Child #3	◯ Female<br>◯ Male
Age of Child #3<br><br>IF CHILD IS LESS THAN 1 YEAR, ENTER 0 WHICH WILL OPEN NEW QUESTION ABOUT MONTHS	______________________<br>(Years)
If Child #3 is less than 1 year of age, how many months old is the child	______________________<br>(Months)
What category best describes Child #3	◯ Sibling<br>◯ Patient<br>◯ Other
Where was Child #3 born?	◯ Home<br>◯ Government Health Facility<br>◯ Government Hospital<br>◯ Private Clinic or Hospital<br>◯ Other
If other, please list here:	____________________________

Did Child #3 sleep under a bed net last night?	◯ Yes<br>◯ No
Has Child #3 stayed overnight in a hospital or clinic for malaria in the past year?	◯ Yes<br>◯ No
Sex of Child #4	◯ Female<br>◯ Male
Age of Child #4<br><br>IF CHILD IS LESS THAN 1 YEAR, ENTER 0 WHICH WILL OPEN NEW QUESTION ABOUT MONTHS	________________<br>(Years)
If Child #4 is less than 1 year of age, how many months old is the child	________________<br>(Months)
What category best describes Child #4	◯ Sibling<br>◯ Patient<br>◯ Other
Where was Child #4 born?	◯ Home<br>◯ Government Health Facility<br>◯ Government Hospital<br>◯ Private Clinic or Hospital<br>◯ Other
If other, please list here:	________________
Did Child #4 sleep under a bed net last night?	◯ Yes<br>◯ No
Has Child #4 stayed overnight in a hospital or clinic for malaria in the past year?	◯ Yes<br>◯ No
Sex of Child #5	◯ Female<br>◯ Male
Age of Child #5<br><br>IF CHILD IS LESS THAN 1 YEAR, ENTER 0 WHICH WILL OPEN NEW QUESTION ABOUT MONTHS	________________<br>(Years)
If Child #5 is less than 1 year of age, how many months old is the child	________________<br>(Months)
What category best describes Child #5	◯ Sibling<br>◯ Patient<br>◯ Other

Where was Child #5 born?	◯ Home<br>◯ Government Health Facility<br>◯ Government Hospital<br>◯ Private Clinic or Hospital<br>◯ Other
If other, please list here:	__________________________________
Did Child #5 sleep under a bed net last night?	◯ Yes<br>◯ No
Has Child #5 stayed overnight in a hospital or clinic for malaria in the past year?	◯ Yes<br>◯ No
Sex of Child #6	◯ Female<br>◯ Male
Age of Child #6<br><br>IF CHILD IS LESS THAN 1 YEAR, ENTER 0 WHICH WILL OPEN NEW QUESTION ABOUT MONTHS	___________________________<br>(Years)
If Child #6 is less than 1 year of age, how many months old is the child	___________________________<br>(Months)
What category best describes Child #6	◯ Sibling<br>◯ Patient<br>◯ Other
Where was Child #6 born?	◯ Home<br>◯ Government Health Facility<br>◯ Government Hospital<br>◯ Private Clinic or Hospital<br>◯ Other
If other, please list here:	__________________________________
Did Child #6 sleep under a bed net last night?	◯ Yes<br>◯ No
Has Child #6 stayed overnight in a hospital or clinic for malaria in the past year?	◯ Yes<br>◯ No
Additional Comments<br><br>Add information for additional children here (if over 6 children in household)	__________________________________

Household Location and Characteristics

Ask participant about the location and features of the household, and markers of household wealth

Which was the primary mode of transportation you used to travel to St. Paul's Health Center?	◯ Boda boda<br>◯ Car taxi<br>◯ By foot / walking<br>◯ Bus / matatu<br>◯ Other<br>(One that they spent most time traveling)
Which mode of transportation did you use?	__________________________________
How long did it take you to travel to St. Paul's Health Center?	◯ 0 - 30 minutes<br>◯ Over 30 minutes - 1 hour<br>◯ Over 1 hour - 1.5 hours<br>◯ Over 1.5 hours - 2 hours<br>◯ Over 2 hours -3 hours<br>◯ Over 3 hours
Which Parish / ward do you live in?	__________________________________
Which village / cell do you live in?	__________________________________
What is the main source of drinking water for members of your household?	◯ Piped water<br>◯ Tube well or borehole<br>◯ Dug well<br>◯ Water from spring<br>◯ Rainwater<br>◯ Tanker truck<br>◯ Bicycle with jerrycans<br>◯ Surface water (river, lake, stream, irrigation channel)<br>◯ Bottled water<br>◯ Other
If a dug well, please specify the type:	◯ Protected well<br>◯ Unprotected well
Where is the water piped:	◯ Piped into house<br>◯ Piped to yard/plot<br>◯ Piped to neighbor<br>◯ Piped to public tap
If other source of water, please specify	____

Where is that water source located?	◯ In own dwelling<br>◯ In own yard or plot<br>◯ Elsewhere
How many minutes does it take to get there, get water, and come back?	__________________________________<br>(Minutes)
Do you do anything to the water to make it safer to drink?	◯ Yes<br>◯ No
What do you usually do to make the water safer to drink?	☐ Boil<br>☐ Add bleach or chlorine<br>☐ Strain through a cloth<br>☐ Use water filter<br>☐ Solar disinfection<br>☐ Let is stand and settle<br>☐ Other<br>(Water treatment method)
If other method of treating water, please specify:	____
What kind of toilet facility do members of your household usually use?	◯ Flush or pour toilet<br>◯ Pit Latrine<br>◯ Composting toilet<br>◯ Bucket toilet<br>◯ Hanging toilet<br>◯ No facility/bush<br>◯ Other<br>(Toilet type)
If other type of toilet, please specify:	____
Do you share this toilet facility with other households?	◯ Yes<br>◯ No
Including your own household, how many other households use this toilet facility?	__________________________________<br>(# households share toilet)
Where is the toilet located?	◯ In own dwelling<br>◯ In own yard or plot<br>◯ Elsewhere
How many minutes does it take to get to the latrine and come back?	__________________________________<br>(Minutes)

What type of fuel does your household mainly use for cooking?	◯ Electricity<br>◯ LPG/Cylinder Gas<br>◯ Biogas<br>◯ Kerosene<br>◯ Charcoal<br>◯ Wood<br>◯ Straw/Grass<br>◯ Agricultural crop<br>◯ Animal dung<br>◯ No food cooked in household<br>◯ Other<br>(Type of fuel used for cooking)
If other type of fuel, please specify:	__________________________________<br>(Other fuel type)
Where is the cooking of food usually done?	◯ In the house<br>◯ In a separate building<br>◯ Outdoors<br>◯ Other<br>(Cooking location)
If cooking is done somewhere else, please specify:	__________________________________<br>(Cooking location = other)
How many rooms are in this household?	____________________________
How many rooms in this household are used for sleeping?	____________________________
Does this household own any livestock, herds, other farm animals, or poultry?	◯ Yes<br>◯ No
If yes, what type of livestock:	☐ Cattle<br>☐ Goats<br>☐ Sheep<br>☐ Chickens<br>☐ Pigs<br>(Check all that apply)
How many cattle?	____________________________
How many goats?	____________________________
How many sheep?	____________________________
How many chickens?	____________________________

How many pigs?	______________________
Are there any animals that sleep in the house where people sleep?	◯ Yes<br>◯ No
Does your household have:	☐ Electricity<br>☐ Radio<br>☐ Television<br>☐ Computer<br>☐ Refrigerator<br>☐ Casette/CD/DVD player<br>☐ Table<br>☐ Chair<br>☐ Bed<br>☐ Cupboard<br>☐ Clock<br>☐ Sofa set<br>(Check all that apply)
Does any member of this household own:	☐ Watch<br>☐ Mobile phone<br>☐ Bicycle<br>☐ Motorcycle<br>☐ Car or truck<br>(Check all that apply)
Does any member of this household have a bank account, mobile money account, or account with an agent?	◯ Yes<br>◯ No
What is the main material of the floor of your dwelling?	◯ Natural floor - earth/sand<br>◯ Basic floor - wood planks<br>◯ Basic floor - palm/bamboo<br>◯ Finished floor - polished wood<br>◯ Finished floor - concrete<br>◯ Finished floor - tile<br>◯ Finished floor - stones<br>◯ Finished floor - brick<br>◯ Other
If other type of floor, please specify:	____________________________
What is the main material of the roof of your dwelling?	◯ No roof<br>◯ Natural roof - thatch / palm leaf<br>◯ Natural roof - mud<br>◯ Basic roof - mat<br>◯ Basic roof - tin<br>◯ Basic roof - wood planks<br>◯ Basic roof - cardboard<br>◯ Basic roof - plastic tarpaulin<br>◯ Finished roof - iron sheets<br>◯ Finished roof - wood<br>◯ Finished roof - asbestos<br>◯ Finished roof - tiles<br>◯ Finished roof - concrete<br>◯ Finished roof - roofing shingles<br>◯ Other

If other type of roof, please specify:

____

What is the main material of the exterior walls of your dwelling?

- ◯ No walls
- ◯ Natural walls - thatched/straw
- ◯ Natural walls - dirt
- ◯ Basic walls - poles with mud
- ◯ Basic walls - stone with mud
- ◯ Basic walls - unburnt bricks with mud
- ◯ Basic walls - plywood
- ◯ Basic walls - cardboard
- ◯ Basic walls - reused wood
- ◯ Basic walls - unburnt bricks with plaster
- ◯ Basic walls - burnt bricks with mud
- ◯ Finished walls - cement
- ◯ Finished walls - stone with lime/cement
- ◯ Finished walls - burnt bricks with cement
- ◯ Finished walls - cement blocks
- ◯ Finished walls - unburnt bricks with cement
- ◯ Finished walls - wood planks/shingles
- ◯ Other

If other type of walls, please specify:

____

Comments

Please add any additional comments or information about the participant here

____

Intake: History of Present Illness, Labs, Physical Exam

History of Present Illness

Date of symptom onset

____

Date of admission to IPD

____
(Date patient was admitted)

Time of admission to IPD

____

Which of the following symptoms has the child experienced since they became ill?

Fever?
◯ Yes
◯ No
(As reported by caregiver (even if no thermometer was used))

Nausea or poor feeding?
◯ Yes
◯ No

Vomiting?
◯ Yes
◯ No

Headache?
◯ Yes
◯ No

Cough?
◯ Yes
◯ No

Joint or body pain?
◯ Yes
◯ No

Weakness?
◯ Yes
◯ No

Breathing faster than usual?
◯ Yes
◯ No

Impaired consciousness?
◯ Yes
◯ No

Seizures/convulsions?
◯ Yes
◯ No

Other symptoms? (vaginal bleeding, uterine contractions, prostration, not peeing etc.)

____

Since the child became sick, did the caretaker seek treatment anywhere before coming to St Paul's?
◯ yes
◯ no
◯ unsure/unknown

Where?	◯ Government health center<br>◯ Private health center<br>◯ Drug shop<br>◯ VHT<br>◯ Traditional healer (i.e. herbs)<br>◯ Other
Since the child became sick, have they been given any medications or remedies?	◯ Yes<br>◯ No
What have they been given?	______________________________
Has the patient ever been diagnosed with malaria in the past year?	◯ yes<br>◯ no<br>◯ unsure/unknown
Did they receive treatment at that time?	◯ yes<br>◯ no<br>◯ unsure/unknown
What treatment did they receive at that time?	☐ Oral Artemether/Lumefantrine<br>☐ Oral Artesunate/Amodiaquine<br>☐ Dihydroartemisin/ Piperaquine<br>☐ Quinine tablets<br>☐ First line<br>☐ Artesunate<br>☐ IV Quinine<br>☐ Artemether injection<br>☐ Rectal artesunate<br>☐ Other
If other, list here	______________________________
Did they complete the course of that treatment?	◯ yes<br>◯ no<br>◯ unknown
Was the child hospitalized for malaria treatment at that time?	◯ yes<br>◯ no<br>◯ unknown
Did they return to their normal state of health after that treatment? (Did they get completely better?)	◯ Yes<br>◯ No
Additional comments about symptoms or seeking care?	______________________________________
Have you ever been told that the child has any medical problems, including:?	☐ None<br>☐ HIV / ISS<br>☐ Tuberculosis<br>☐ Diabetes<br>☐ High Blood Pressure<br>☐ Other

If other, please specify (list all):	__________________________________
Does the child take any medications every day?	◯ Yes<br>◯ No
Please list the names of all medications that the child takes each day:	__________________________________

Admission Lab Results

Which ward is the patient admitted to?	◯ Pediatric Ward<br>◯ Medical Ward
Blood smear results?	◯ positive<br>◯ negative<br>◯ unknown
What species were visualized in the blood smear?	☐ P falciparum<br>☐ P ovale<br>☐ P viva<br>☐ P malariae<br>☐ other<br>(Check all that apply)
If other list here	____________________________
RDT results?	◯ Positive<br>◯ Negative<br>◯ Unknown
Hemoglobin?	____________________________<br>(g/dl)
White Blood Cell Count?	____________________________<br>(10^9/L)
Platelets?	____________________________<br>(10^9/L)
If any other labs were collected at admission, write the lab name and results here	__________________________________

Medication prescribed upon admission?	◯ No malaria medication given today<br>◯ Oral Artemether/Lumefantrine<br>◯ Oral artesunate/amodiaquine<br>◯ Dihydroartemisin/ Piperaquine<br>◯ Quinine tablets<br>◯ First line<br>◯ Artesunate<br>◯ IV Quinine<br>◯ Artemether injection<br>◯ Rectal artesunate<br>◯ Other
If other list here	__________________________

Physical Exam?

How old is the child?	◯ Younger than 5 years<br>◯ 5 to 17 years of age
MUAC?	__________________________<br>(What is the measurement of child's mean upper arm circumference? In CM?)
Who collected the MUAC?	◯ Medical team at St. Paul's while doing rounds<br>◯ Severe Malaria Project team, because they did not collect this data during rounds<br>◯ Severe Malaria Project team, because the data recorded during rounds was incorrect / impossible
Eye-Opening	◯ Spontaneous<br>◯ To Speech<br>◯ To Pain<br>◯ No response
Best Motor Response	◯ Obeys<br>◯ Localizes<br>◯ Withdraws<br>◯ Abnormal Flexion<br>◯ Extensor Response<br>◯ No response
Verbal Response:	◯ Orientated<br>◯ Confused conversation<br>◯ Inappropriate words<br>◯ Incomprehensible sounds<br>◯ No response
Total GCS Score	__________________________
Best motor response:	◯ Localizes to painful stimulus<br>◯ Withdraws limb from painful stimulus<br>◯ No response or inappropriate response

Best verbal response:	◯ Cries/speaks appropriately with painful stimulus<br>◯ Moan or abnormal cry with painful stimulus<br>◯ No vocal response to painful stimulus
Eye movements:	◯ Watches or follows<br>◯ Fails to watch or follow
Total Score:	__________________________________
Comments on intake / physical exam:	____

Daily Inpatient Form

Study ID Number ______________________

Patient name ______________________

Date ______________________

Enter the first recorded vitals taken for this participant on this day:

Pulse ______________________
(beats/min)

Who collected the pulse?
- ◯ Medical team at St. Paul's while doing rounds
- ◯ Severe Malaria Project team, because they did not collect this data during rounds
- ◯ Severe Malaria Project team, because the data recorded during rounds was incorrect / impossible

Oxygen Saturation ______________________

Who collected the oxygen saturation?
- ◯ Medical team at St. Paul's while doing rounds
- ◯ Severe Malaria Project team, because they did not collect this data during rounds
- ◯ Severe Malaria Project team, because the data recorded during rounds was incorrect / implausible

Temperature ______________________
(in degrees Celsius)

Who collected the temperature?
- ◯ Medical team at St. Paul's while doing rounds
- ◯ Severe Malaria Project team, because they did not collect this data during rounds
- ◯ Severe Malaria Project team, because the data recorded during rounds was incorrect / implausible

Blood pressure ______________________

Who collected the blood pressure?
- ◯ Medical team at St. Paul's while doing rounds
- ◯ Severe Malaria Project team, because they did not collect this data during rounds
- ◯ Severe Malaria Project team, because the data recorded during rounds was incorrect / implausible

Eye-Opening
- ◯ Spontaneous
- ◯ To Speech
- ◯ To Pain
- ◯ No response

Best Motor Response	◯ Obeys<br>◯ Localizes<br>◯ Withdraws<br>◯ Abnormal Flexion<br>◯ Extensor Response<br>◯ No response
Verbal Response:	◯ Orientated<br>◯ Confused conversation<br>◯ Inappropriate words<br>◯ Incomprehensible sounds<br>◯ No response
Total Score	__________________________
Best motor response:	◯ Localizes to painful stimulus<br>◯ Withdraws limb from painful stimulus<br>◯ No response or inappropriate response
Best verbal response:	◯ Cries/speaks appropriately with painful stimulus<br>◯ Moan or abnormal cry with painful stimulus<br>◯ No vocal response to painful stimulus
Eye movements:	◯ Watches or follows<br>◯ Fails to watch or follow
Total Score:	__________________________
Physical Exam Findings today?	☐ pallor<br>☐ jaundice<br>☐ signs of dehydration (tenting/turgor)<br>☐ other<br>☐ none<br>☐ Unknown/ not recorded
Note other physical exam findings here	________________________________
Has the child experienced any of the following today?	☐ Respiratory distress<br>☐ Convulsion<br>☐ Change in consciousness<br>☐ Spontaneous bleeding<br>☐ Other<br>☐ None<br>☐ Unknown/ not recorded
If other, please describe:	__________________________

Medications used today:	◯ No malaria medication given today<br>◯ Oral Artemether/Lumefantrine<br>◯ Oral artesunate/amodiaquine<br>◯ Dihydroartemisin/ Piperaquine<br>◯ Quinine tablets<br>◯ First line<br>◯ Artesunate<br>◯ IV Quinine<br>◯ Artemether injection<br>◯ Rectal artesunate<br>◯ other/unknown
Has the child received at least 3 doses of IV Artesunate?	◯ Yes<br>◯ No<br>(3+ doses of IV artesunate)
In addition to antimalarial medications, did the patient receive any supportive care today?	◯ yes<br>◯ no<br>◯ unknown
What kind of supportive care did the patient receive?	☐ normal saline<br>☐ Ringer's lactate<br>☐ dextrose<br>☐ oxygen via nasal prongs<br>☐ blood transfusion<br>☐ IV metronidazole<br>☐ IV ciprofloxacin<br>☐ IV ceftriaxone<br>☐ IV gentamicin<br>☐ IV ampicillin<br>☐ zinc<br>☐ paracetamol<br>☐ other
If other supportive care was given, note that here	________________________________
DISCHARGE<br><br>What medications were prescribed upon discharge?	☐ Coartem<br>☐ D-Artepp<br>☐ ORS<br>☐ Paracetamol<br>☐ Zinc<br>☐ Omeprazole<br>☐ Other
What other medications were prescribed upon discharge?	________________________________
Labs collected today?	◯ Yes<br>◯ No
Blood smear results?	◯ not collected<br>◯ positive<br>◯ negative<br>◯ other/unknown

Hemoglobin?

____
(g/dl)

White Blood Cell Count?

____
(10^9/L)

Lym% - lymphocytes

____
(lym%)

Gran% - Granulocytes

____
(gran%)

Mid%

____
(mid%)

Lym# - lymphocytes #

____
(lym#)

Gran # - Granulocytes #

____
(gran#)

Mid#

____
(mid#)

Platelets?

____
(10^9/L)

Was a Liver Function Test done?
○ Yes
○ No

ALP (Liver)

____
(U/L)

ALT (Liver)

____
(u/L)

AST (Liver)

____
(U/L)

Alb (Liver)

____
(g/dl)

IBiL (Liver)

____
(mol/l)

DBiL (Liver)

____
(umol/l)

TP (Liver)

____
(mg/dl)

If any other labs were collected today, write the lab name and results here:

____

Additional diagnosis?

Has the patient received any other diagnosis besides malaria during the current illness? If yes, please specify the additional diagnosis.

____

Disposition

- ◯ Remains in IPC
- ◯ Discharged home
- ◯ Transferred to other facility
- ◯ Deceased
- ◯ left against medical advice
- ◯ unknown/other

What was the date of death of the patient?

____

Date of discharge

____

What time was the patient discharged?

____

Additional comments?

____

Malaria Posthospitalization Day 14

Patient name

____

Date of follow-up survey

____

Did the follow-up survey take place 14 days after discharge?

- ◯ Yes
- ◯ No

How many days after discharge did the follow-up survey take place?

____

Symptoms

Which best describes the participant's vital status?

- ◯ Outpatient
- ◯ Hospitalized
- ◯ Deceased

Express sympathy for the death of the child. Ask if it is alright to continue with the survey or if the caregiver would prefer to end the conversation at this time.

Ok to proceed with the survey?

- ◯ Yes
- ◯ No

Does the caregiver believe the child's health has returned to normal?

- ◯ Yes
- ◯ No
- ◯ Caregiver doesn't know

Is the patient currently having symptoms related to their hospitalization?

- ◯ Yes
- ◯ No

Symptoms Present

- ☐ Fever
- ☐ Nausea / Not feeding
- ☐ Headache
- ☐ Body ache
- ☐ Runny nose
- ☐ Cough

If the patient is experiencing any of the following symptoms, please indicate whether the symptom has improved, remained the same, or become worse. if the patient is not experiencing a symptom, please select "none".

	None	Improved	About the same	Worse
Fever	◯	◯	◯	◯
Headache	◯	◯	◯	◯
Lethargy	◯	◯	◯	◯
Seizures	◯	◯	◯	◯

Nausea / Not feeding	◯	◯	◯	◯
Vomiting	◯	◯	◯	◯
Abnormal behaviors	◯	◯	◯	◯
Altered mental status	◯	◯	◯	◯
Body ache	◯	◯	◯	◯

Is the patient experiencing any other symptoms?
◯ Yes
◯ No

Please describe these other symptoms:
____

Are any of the symptoms reported above newly developed since hospital discharge?
◯ Yes
◯ No

Which symptoms are newly developed?
☐ Fever
☐ Nausea / Not eating
☐ Headache
☐ Body ache
☐ Runny nose
☐ Cough
☐ Seizures
☐ Vomiting
☐ Behavioral abnormality
☐ Altered mental status
☐ Other

If other, please describe
____

Comments on symptoms
____

Care Seeking

Have you taken the child for additional care in the past 2 weeks?
◯ Yes
◯ No

Where did you seek services in the past two weeks?
☐ Government health center
☐ Private health center
☐ Drug shop
☐ VHT
☐ Traditional healer (i.e. herbs)
☐ Other

If the caregiver sought care elsewhere, please specify:
____

Please describe the reasons for seeking care
____

Were any treatments or medications prescribed?	◯ Yes<br>◯ No
If yes, please list them here:	__________________________________

Medication Use

Is the patient still taking any medications prescribed at hospital discharge?	◯ Yes<br>◯ No
If yes, please list the medication names and dosages here:	__________________________________
Is the patient taking any newly prescribed medications?	◯ Yes<br>◯ No
If yes, please list the medication names and dosages here:	__________________________________
Has the patient received any malaria testing since hospitalization?	◯ Yes<br>◯ No
What was the diagnosis?	◯ Malaria confirmed<br>◯ Malaria not confirmed

Comments

Is there anything else you would like to share about the patient's health since hospitalization?	◯ Yes<br>◯ No
Please describe:	__________________________________
Additional comments?	__________________________________
