# Supplementary material for: Quality of care and post-discharge morbidity among children diagnosed with severe malaria in rural Uganda: A prospective cohort study
Source: PLOS Glob Public Health. 2024 Oct 7;4(10):e0003794. doi: 10.1371/journal.pgph.0003794 (PMC11458001; doi:10.1371/journal.pgph.0003794)
Supplement: Attachment — Submitted filename: Response to Reviewers_PLOS Global Public Heatlh (29JUL24).docx [file pgph.0003794.s003.docx]

July 26, 2024

Dr. Amy Kristine Bei

Academic Editor
*PLOS Global Health*

RE: PGPH-D-24-01221 – Response to reviewer comments

Dear Dr. Bei:

Thank you for the review of our manuscript entitled, **“Quality of Care and Post-Discharge Morbidity Among Pediatric Severe Malaria Patients in a Rural Area of Uganda: A Prospective Cohort Study.”** We appreciate the detailed review and believe that the revised manuscript is much improved. Please find our responses (in blue) to reviewers’ comments below:

**REVIEWER 1**

**Overall**: Reviewer #1: This is a pretty straight forward description of a study investigating the in-facility treatment and post-discharge outcomes of children presenting to a lower-level health centre in Uganda with features of severe malaria. The paper is generally well written and referenced and the statistics seem appropriate and nicely summarised in sensible tables.

**Response**: Thank you, we appreciate your feedback.

**Major Comment 1.1**: The title is a bit confusing. I was expecting to read a paper that was aimed at investigating the association between the quality of care received in the health facility and the post-discharge outcome. It soon became apparent that >80% of the children received treatment that complied with WHO standards and that the authors made no attempt to relate care-quality to outcome. I suggest a change to the title!

**Response**: We appreciate the reviewer’s sentiment. We also did not expect the quality of care to be so high and at the same time did not anticipate the relatively high prevalence of persistent symptoms. We note that we do present the association between care-quality (compliance with key components of the WHO guidelines) and post-discharge outcomes in **Table 4**, although we did not find an association, likely due to the small number of individuals who received sub-optimal care.

To reduce confusion, we removed the word “high” from the title to remove any descriptors and make it clearer that we were not specifically investigating that association.

**Major Comment 1.2**: The post-discharge morbidities recorded were rather soft and subjective. Some (such as "not feeling well") could apply to any child who was not completely back to normal (which would seem unlikely in many children who had been admitted to hospital with a life-threatening condition only 2 weeks after discharge). I wonder how standard the methods and definitions are?

**Response**: First, we wish to highlight that “not feeling well” was not one of our post-discharge symptoms. Perhaps the reviewer misread not **feeding** well, which we believe is a more specific outcome measure. However, we agree with the overall sentiment that some of the post-discharge morbidities, such as not feeding well and fever, are subjective given that they are based on caregiver reporting. We again note that we did not expect to find this level of post-discharge morbidities and the original study design and focus was on the quality of care received in clinic.

In response to this comment, we have added the following sentence in the limitations section to address the subjectivity of certain post-discharge symptoms:

*Moreover, certain caregiver-reported symptoms collected at the 14-day follow-up call were subjective, such as fever and not feeding well, which could introduce misclassification of the outcome.*

**Major Comment 1.3**: In the results section and in Figure 1, the authors present data on the number of patients with suspected or confirmed malaria in Kasese District between July and December 2023. However, these data don’t seem to be derived from this study and there is no description of the methods used in the methods section. If not part of the study then they should not be included in the results but referred to (with an appropriate citation) either in the introduction or discussion. They could potentially remain in the figure for comparative purposes with an appropriate foot note and reference.

**Response**: We point the reviewer to line 139 in the methods section where we state: “We obtained aggregated data on district level malaria cases from the Kasese District Health Department.”

In response to this comment, we have added to the methods that this data was derived from the HMIS system from the Kasese District Health Department.

**Major Comment 1.4**: The authors should probably be more cautious in their interpretation of persisting parasitaemia beyond 24 hours. Although most will clear parasites within that time, this is not universal, even in the era before resistance (see for example White Malar J (2017) 16:88).

**Response**: We agree that we do not want to make this assumption, we only say that it’s a possibility that warrants future research. These 6 participants were tested after 2+ days of receiving artesunate, tested positive a second time, and then were switched to quinine. Given that we do not have timestamps on when the artesunate was given on a particular day, we state 24 hours to be conservative although it was likely longer than this for most participants. No changes made.

**Major Comment 1.5**: The authors could also be a bit more clear about the fact that this group of children probably included a significant number who didn't actually have severe malaria but were suffering instead from another serious illness with incidental parasitaemia (see for example Watson et al., Sci. Transl. Med. 14, eabn5040 (2022 and Watson, Ndila, et al. eLife 2021;10:e69698). They do mention it briefly in the discussion but I think they could be more upfront about it.

**Response**: In response to this comment, we have made changes to the manuscript in two places.

We added the following information in the results section to provide context that some children did have additional diagnoses:

*Many patients (117, 64.6%) had a second diagnosis recorded at SPHC, with the most common including septicemia and anemia with no underlying diagnosis of sickle cell disease (SCD) – both of which may be associated with malaria - as well as pneumonia, and SCD.*

We also added the following context to the limitations section:

*In addition, many patients had other diagnoses in addition to malaria. Therefore, the severity of illness in some children may have been primarily caused by another serious illness with incidental parasitemia rather than by severe malaria.*

**Major Comment 1.6**: Incidentally, do the authors have any other data that would help them tease apart the previous point?

**Response**: Please see our response to Major Comment 1.5, where we elaborate on secondary diagnoses within the cohort.

**Minor Comment 1.1**: In the abstract, the authors state that there were 6 deaths recorded by the 14-day post-discharge follow-up. This is a bit deceptive because 3 of the deaths occurred in hospital and only 3 occurred post discharge. Please clarify in the abstract.

**Response**: We clarified this in the abstract as follows:

*Six deaths were recorded, including three at SPHC and three post-transfer or discharge.*

**Minor Comment 1.10**: Reference 5 should be replaced with the original citation rather than a citation to the WHO guideline.

**Response**: We have changed this citation to include the original study citations.

**REVIEWER 2**

**Overall**: This was a well-written manuscript of an observational longitudinal cohort study evaluating care and adherence to WHO guidelines for pediatric severe malaria cases. The study took place at a private health facility in Kasese, Uganda during the end of the dry season into the rainy season. Participants were followed from admission to discharge and contacted 14 days post-discharge to assess for any symptom onset. This was a very interesting study and I appreciate the authors’ thorough description of the epidemiology of malaria in the district in addition to the findings from the cohort. I have a few comments for the author’s consideration.

**Response**: Thank you.

**Methods**

**Comment 2.1**: Eligibility criteria section - Could authors confirm whether the eligibility criteria included only those with severe or complicated malaria or did it include all children? Eligibility criteria seems like any confirmed case was eligible.

**Response**: The eligibility criteria includes all children who were admitted to SPHC and who were being treated for malaria (either confirmed or expected). The study did not include outpatients nor inpatients who were not treated for malaria. To make this clearer, we added details to the exclusion criteria as follows:

*We excluded outpatients, inpatients not being treated for malaria, and pregnant patients, as they would be admitted to a separate ward.*

**Comment 2.2**: Statistical analysis: “We ran a generalized linear model with a log link function to assess crude and adjusted risk ratios (RRs) for the binomial outcome of experiencing symptoms 14 days post-discharge.” - Perhaps authors can clarify here what the research question was here (e.g., assess predictors of symptom onset post-discharge)?

**Response**: Thank you. We have updated the language as follows:

*To assess associations between factors related with hospitalization and the presence of symptoms 14 days post-discharge, we ran a generalized linear model with a log link function and present crude and adjusted risk ratios (RRs).*

**Results**

**Comment 2.3**: Line 241 - Could authors specify what treatments the participants who did not receive 3+ doses of IV artesunate or ACT?

**Response**: On line 222, where we describe that 186 out of 187 received IV antimalarial medication, with the percentages that received IV artesunate and IV quinine. All participants (100%) received either IV artesunate or ACTs.

**Comment 2.4**: Table 1 - Regarding “Received treatment or medication prior to admission” and “Type of medication received” - Do authors mean treatment/medication to refer to any medications or specific to treatment of malaria?

**Response**: “Received treatment or medication prior to admission” refers to patients who received any treatment or medication, whether or not for malaria. Under “Type of medication received”, we specify the percentage of those who received medication that received first-line anti-malarial medication, ineffective medication, or the caregiver did not know what kind of medication they received. In response to this comment, we have added another row to Table 1 with data for “Non-antimalarial only” medication.

**Comment 2.5**: Table 2 “Received 3+ Doses of IV Artesunate followed by Prescription of ACTs” sub-section - It might be more informative if authors could provide the n (%) for those who received 3+ dose IV only, and those who received ACT prescriptions only.

**Response**: In response to this comment, we have changed the categories to the following: 3+ doses IV artesunate and ACT; ACT, no 3+ doses IV artesunate; and 3+ doses artesunate, no ACTs.

**Comment 2.6**: Table 2 - Authors may consider adding a footnote that blue indicates compliance in the “a” footnote.

**Response**: We have added the following text in the “a” footnote of Table 2:

*^“^Blue rows indicate compliance to WHO guidelines.”*

**Comment 2.7**: Line 211 - “peaked” rather than “peeked”

**Response**: Corrected.

**Comment 2.8**: Fig 1 - Could authors provide trends in severe malaria cases to reference their statement?

**Response**: We’re not sure what statement the reviewer is referencing. We changed the sentence in line 216 to specify that “The greatest number of severe malaria cases ***admitted to SPHC*** peaked earlier, at the beginning of the rainy season in September” to clarify the severe malaria cases.

**Comment 2.9**: Line 265 - Medication for treatment of malaria or any medication?

**Response**: In line 265, participants reported that they were taking any newly prescribed medication, not just for the treatment of malaria. In response to this comment, we added the proportion that specifically stated taking antimalarial medication to Table 3, and we added the following text in bold to line 270 in the results section.

*A substantial proportion of participants (17.5%) reported that they were taking newly prescribed medication since being discharged,* ***with 8 (4.5%) reporting newly prescribed antimalarial medication.***

**Comment 2.10**: Line 275-277 - Could authors also describe what proportion of those who experienced altered consciousness received IV artesunate + ACTs?

**Response**: 71 (93.4%) participants who experienced altered consciousness overall, and 67 (97.1%) with altered consciousness excluding the patients who died at SPHC or were transferred to another facility, received 3+ doses of IV artesunate and ACTs. Because this treatment rate is similar to the overall population described, we did not break it out specifically in the manuscript.

**Comment 2.11**: Could authors report p-values, only for the sake of understanding how authors decided which variables when into the adjusted model.

**Response**: We refer the reviewer to the methods section, line 167, where we note that “We included variables with a crude estimate p-value of less than 0.2 in the adjusted model.” In response to this comment, we added the specific p-values for all variables included in the model in the “c” footnote of Table 4 as follows:

*Adjusted risk ratio model included all variables with a crude estimate p-value of less than 0.2. This model included care seeking prior to admission (p = 0.16), altered consciousness (p = 0.05), and respiratory distress (p=0.14)*

**Comment 2.12**: Table 4 - Authors could consider adding n (%) for the outcome in addition to the RRs to understand the proportions of those who experienced each symptom.

**Response**: We have added a column with n (%) in Table 4. We also added the following text as the “b” footnote:

*Column presents the number and percentage of participants in each category who reported having symptoms 14-days post-discharge*

**Discussion**

**Comment 2.12**: Line 291 - Wasn’t this 81.3% not 82.2% as per Table 2?

**Response**: Thank you – we have double checked our calculations and corrected these percentages.

**Comment 2.12**: I think a major comment would be that authors could consider adding some discussion around the recent 2022 WHO guidelines regarding post-discharge malaria chemoprevention. One of the authors’ main points is that a high proportion of participants are experiencing symptoms mainly fever. While symptom classification was based on self-report, it is possible (though unknown) that children were re-infected or experienced recrudescence.

**Response**: We appreciate this feedback and have added the following to the discussion at line 345:

*Given the potential for re-infection, implementing intermittent post-discharge chemoprevention, as recommended in the 2023 WHO Malaria Guidelines, may reduce post-discharge morbidity and should be investigated further [5].*

**Comment 2.12**: Line 294 - I think it’s important to report what proportion of children received IV artesunate only without a course of ACT. IV artesunate will rapidly reduce parasite burden, but it has a fairly short half-life and thus needs to be followed with ACTs to clear residual parasitemia. While I expect this # to be small, IV artesunate without follow up of oral ACT is concerning re: drug resistance and recrudescence.

**Response**: We added this data to the Results section, line 247, to report the percentage of children who received IV artesunate but not ACTs:

*Among 177 participants who were discharged from SPHC, excluding those who were transferred to another facility, left against medical advice, or died while at SPHC, two (1.1%) received 3+ doses of IV artesunate but did not receive ACTs.*

**Comment 2.12**: Did these 6 children also receive ACTs following IV artesunate? And how long after were these children tested? I don’t see this in the results section, but maybe I missed it? This might be a good section where authors could discuss post-discharge malaria chemo prevention.

**Response**: While we are not certain which children the reviewer is referring to (6 children who died or 6 who tested positive after received IV artesunate), we assume they may be referring to line 342. We added “and then were switched to quinine” to clarify that these were the 6 children who started on IV artesunate and then were switched to IV quinine after testing positive a second time after being on IV artesunate.

We refer the reviewer to line 224 where we provide information on the testing timelines: *In 6 cases (3.2%), participants tested positive on a blood smear at least 24 hours after initial treatment with artesunate, and were then switched to IV quinine.*

All 6 of these children did receive ACTs.

**Comment 2.12**: Limitations - RE: caregiver reporting - I think this is more generally a problem with misclassification of the outcome rather than recall bias. For example, fever is subjective.

**Response**: We agree with your comment, although we think recall bias could also be a possibility. For example, upon intake, caregivers of certain patients may misremember the specific day the child started feeling ill, or the type of medication received prior to arrival at SPHC.

We agree that with the 14-day follow-up symptoms, misclassification would be a concern of subjective symptoms such as fever since we are asking about current patient symptoms. We have added to the text in the limitations as follows:

*Additionally, data on history of illness, including questions about the specific day when the child started feeling ill and medications received before arriving at SPHC, were based on caregiver reporting, which could introduce recall bias. Moreover, certain caregiver-reported symptoms collected at the 14-day follow-up call were subjective, such as fever and not feeding well, which could introduce misclassification of the outcome.*

Thank you for your continued consideration of our manuscript.

Kind regards,

Ross M. Boyce MD, MSc

Assistant Professor

Division of Infectious Diseases
