# Supplementary material for: Quality of care and post-discharge morbidity among children diagnosed with severe malaria in rural Uganda: A prospective cohort study
Source: PLOS Glob Public Health. 2024 Oct 7;4(10):e0003794. doi: 10.1371/journal.pgph.0003794 (PMC11458001; doi:10.1371/journal.pgph.0003794)
Supplement: Attachment — Submitted filename: R2R2_PLOS GPH (12SEP24).docx [file pgph.0003794.s004.docx]

September 12, 2024

Dr. Amy Kristine Bei

Academic Editor
*PLOS Global Health*

RE: PGPH-D-24-01221

Dear Dr. Bei:

Thank you for the review of our manuscript. We appreciate the additional comments. Please find our responses (in blue) below:

**Academic Editor**

**Comment 1**: Table 4 - Please revise “RR” column to “Crude RR”

**Response**: Change made

**Comment 2**: The study eligibility criteria was inpatient children of pediatric and general wards with confirmed or suspected malaria. Thus, this cohort of children included those with and without severe malaria diagnosis. In fact, authors state that only 62% of children had a symptom consistent with severe malaria (lines 191-192). I see that authors responded to Reviewer #1’s comment regarding this point (Major Comment 1.5), but I agree that in this revised version, authors should be more upfront about this point throughout the manuscript, including a change to the title which states this is a study of pediatric severe malaria cases (which is not necessarily the case). Moreover, authors could consider adding “severe malaria diagnosis” as a risk factor for patient outcomes 14-days post-discharge."

**Response**: We readily acknowledge that there is uncertainty in confirming any diagnosis without the aid of more diagnostics, especially when attempting to differentiate between (i) severe malaria and (ii) other infections (e.g., bacteremia, influenza, etc.) in which *P. falciparum* parasitemia may also be present. We highlight that ~95% of participants did have a positive mRDT or smear on admission. We also note that while only 62% of children had documentation of a symptoms consistent with severe malaria, measurement relied on the physical examination performed by the attendant clinician. We have previously shown that clinical examination alone (i.e., without testing for other manifestations of severe malaria like lactic acidosis) frequently fails to identify a large proportion of cases (Boyce et al., *Clin Inf Dis*., 2016. Therefore, we caution against assuming that the remaining 38% of participants did not have severe malaria just because a defining symptom was not identified on physical examination.

Regardless, we appreciate the limitation of our study methods and agree that a change in the title to more accurately reflects the uncertainty is appropriate. The title is now:

**Quality of Care and Post-Discharge Morbidity Among Children Diagnosed with Severe Malaria in Rural Uganda: A Prospective Cohort Study**

Thank you for your continued consideration of our manuscript.

Kind regards,

Ross M. Boyce MD, MSc

Assistant Professor

Division of Infectious Diseases
